# An improved protocol for carrot haploid and doubled haploid plant production using induced parthenogenesis and ovule excision *in vitro*

**DOI:** 10.1007/s11627-014-9597-1

**Published:** 2014-03-08

**Authors:** Agnieszka Kiełkowska, Adela Adamus, Rafal Baranski

**Affiliations:** Institute of Plant Biology and Biotechnology, Faculty of Horticulture, University of Agriculture in Krakow, Al. 29-Listopada 54, 31-425 Krakow, Poland

**Keywords:** *Daucus carota*, Doubled haploid, Wide pollination, Ovule culture

## Abstract

In this work, we describe an improved protocol for induced parthenogenesis and ovule culture of carrot (*Daucus carota* L.). The effects of pollination with parsley pollen and/or 2,4-dichlorophenoxyacetic acid (2,4-D) treatment on the stimulation of parthenogenesis were studied using heterozygous donor plants of 30 varieties and breeding populations of carrots. Isolated ovules, cultured *in vitro*, enlarged and developed embryos or calli. The application of 2,4-D on pollinated flowers stimulated callus development but did not increase the frequency of embryo development from ovules and, thus, was not useful for increasing the frequency of haploid plant recovery. The efficiency of embryo development was accession-dependent and varied from 0 to 24.29%. In optimized conditions, most accessions responded by embryo development exclusively. The highest frequency of embryo development was observed from ovules excised from ovaries 20–22 d after pollination with parsley pollen. Among several media used for ovule culture, 1/2-strength Murashige and Skoog medium with 0.06 μM indole-3-acetic acid (IAA) was the best. It allowed the production of embryos at a similar frequency as on the media supplemented with kinetin, gibberellic acid, putrescine, or thidiazuron, but restricted callus development. Most plants obtained were haploids and diploids derived from parthenogenesis, as evidenced by homozygosity at three independent loci based on isozyme and PCR analyses. In total, considering haploids and embryo-derived homozygous diploids together, 72.6% of regenerated plants were of gametic origin.

## Introduction

Carrot is one of the top ten most economically important vegetables in the world (FAO 2010). It is an outcrossing, insect-pollinated diploid (2n = 2x = 18). Genetic improvement of cultivated carrot has focused on root yield, morphology, and quality, *i.e.*, root shape and size, skin color, phloem and xylem color, skin surface, core diameter, carotenoid, and sugar contents (Rubatzky et al. 1999, Baranski et al. 2012). The discovery and application of genetic-cytoplasmic male sterility (CMS) systems opened new perspectives in carrot breeding (Simon et al. 2008), and the development of F_1_ hybrid varieties has relied on the use of CMS lines. Hybrid varieties account for the majority of the carrot seeds sold in the world today and are produced using inbred populations obtained after several generations of self- or sib-pollination that also results in strong inbreeding depression. As carrot is a biennial crop, the development of highly homozygous population is time-consuming (Stein and Nothnagel 1995).

The use of completely homozygous doubled haploid (DH) lines would considerably shorten breeding processes. Haploids and DH lines can be obtained in various ways, including anther, microspore, and ovule culture, and DH lines have been used successfully in breeding programs of many crops, including barley, wheat, maize, pepper, rice, and tobacco (Thomas et al. 2003). Most production systems utilize highly efficient androgenesis from cultured anthers or isolated microspores. Studies on anther culture in carrots showed that most of the regenerated plants were of somatic origin, caused by frequent indirect embryogenesis and callus formation from anthers (Adamus and Michalik 2003, Staniaszek and Habdas 2006). Culture of isolated microspores in liquid media has also been reported, but the frequency of carrot haploid development *via* direct embryogenesis from microspores was low (Matsubara et al. 1995). Secondary embryogenesis, occurring from both primary embryos and calli, increased the final number of regenerants and resulted in the regeneration of plants of various ploidies, including haploids, diploids, and triploids. The response from isolated microspore cultures was highly dependent on the donor plant genotype (Li et al. 2013).

Ovule or ovary cultures are an alternative method for haploid production. Haploid production from the female gametophyte occurs *via* either gynogenesis or parthenogenesis induced by wide pollination. In gynogenesis, haploids arise from an unfertilized egg cell that is stimulated to divide by donor plant pretreatment and *in vitro* culture conditions (Bohanec et al. 1995). In parthenogenesis, haploids develop as a result of stimulation by application of irradiated pollen or pollen of other species or genera (wide pollination; Foroughi-Wehr and Wenzel 1993). Parthenogenesis induced by wide pollination was reported for *Brassica* sp. (Eenink 1974), *Pisum sativum* (Virk and Gupta 1984), and *Gossypium hirsutum* (Sacks 2008, Kantartzi and Roupakias 2009). Parthenogenesis has also been described for carrots (Kiełkowska and Adamus 2010) after stimulation of ovule development by pollination by other *Apiaceae* species like parsley or celery. Haploid and diploid plants were obtained from unfertilized ovules cultured *in vitro*. The efficiency of this laborious and time-consuming process was low: only 1.85% of cultured ovules responded and more than half of them showed callus development rather than embryo development. Thus, the usefulness of the method was limited.

In the present study, we examined the effects of modifications of the conditions used for *in vitro* culture of carrot ovules in increasing the frequency of embryo development and validated the results using a wide selection of carrot accessions.

## Materials and Methods

For each year of the study (2008–2011), vernalized roots of carrot accessions (cultivars and breeding lines) and parsley were planted in pots containing a mixture of peat moss and coarse sand (1:1 v/v) and were grown in the greenhouse. Each carrot plant was verified to be heterozygous at three loci. For these assays, young leaves were collected and used to detect isozyme variants of glucose-6-phosphate isomerase (PGI, EC 5.3.1.9) according to Baranski (2000). Plant DNA was isolated from the same leaves according to the method by Rogers and Bendich (1988). Polymerase chain reaction (PCR) was used to amplify regions of the *chs2* (chalcone synthase) and *ipi3* (isopentenyl diphosphate isomerase) genes using these primer pairs (5′–3′): chs2 — CTC AAG GAG AAG TTT AGG CGG ATG and ATG AGG CCA TGT ACT CGC AGA AAC; and ipi3 — CTG TAC AGG GAG TCC GAG CTT ATC and CCA ATC CAA GAC ATT TAC CAT AGG TC.

Each PCR reaction mixture (10 μl) contained 1 μl 10× Taq buffer, 0.8 μl 25 mM MgCl_2_, 0.25 μl 10 mM dNTPs, 0.5 μl 10 μM primer, 0.1 μl Taq DNA polymerase (5 u/μl, Fermentas, ABO, Gdańsk, Poland), and 1 μl (10–20 ng) template DNA. The thermal cycler (Eppendorf Master Gradient, Eppendorf Scientific, Inc., Westbury, NY) was programmed for 2 min at 94°C, 30 cycles of 30 s at 94°C, 30 s at 55°C, 3 min at 68°C, and a final 10 min extension at 68°C. Amplified DNA fragments were separated in 1% agarose gels containing ethidium bromide and visualized in UV light. The amplified fragments had sizes of approximately 820 or 900 bp for *chs2* and approximately 1,050 or 1,100 bp for *ipi3*. The presence of a single band for a given locus was considered evidence of homozygosity at that locus, while the presence of two bands was considered evidence of heterozygosity. Plants that were heterozygous for at least two of three (*pgi*-*2*, *chs2*, or *ipi3*) loci were selected and used in further experiments as ovule donors.

At the beginning of flowering, the plants were transferred to netted isolation cages. Hand pollination of carrot umbels with parsley pollen was performed after stamens fell and stigmas were receptive. A subset of umbels (approximately 50%) were sprayed with 450 μM dichlorophenoxyacetic acid (2,4-D) 24 h after pollination (treatment PS), while the rest were not (treatment PU). Additionally, two controls were used: unpollinated umbels sprayed as described above with 2,4-D (treatment US) and unpollinated and unsprayed umbels (treatment UU).

Each umbel from treatments PS, PU, and UU was kept untouched on the plants in the greenhouse in netted isolation cages to allow natural seed set. The remaining umbels were harvested 7 d, 14–16 d, or 20–22 d after pollination (DAP), surface sterilized in 70% ethanol for 5 min, transferred to 10% chloramine T (Bochemie Poland, Katowice, Poland) for 20 min, and washed three times with sterile distilled water for 5 min. Ovules were isolated from enlarged ovaries under sterile conditions and placed in 60-mm Petri dishes containing 5 ml medium. Explants were cultured on one of the following media: H — according to Kiełkowska and Adamus (2010) with 0.06 μM indole-3-acetic acid (IAA); O — 1/2-strength Murashige and Skoog (MS) (Murashige and Skoog 1962) with 100 mM MnSO_4_, 4.16 mM Edamin K (Sigma Aldrich Ltd., Poznań, Poland),) and 30 g l^−1^ sucrose; SHI — full-strength MS with B5 vitamins (Gamborg et al. 1968) and 0.57 μM IAA, 1.44 μM gibberellic acid (GA_3_), 4.6 μM kinetin, and 40 g l^−1^ sucrose; SHT — SHI medium with 9.08 μM thidiazuron (TDZ); HT — H medium with 9.08 μM TDZ; HP — H medium with 1 mM putrescine; or SHP — SHI medium with 1 mM putrescine. All media contained 7 g l^−1^ Lab-agar (Biocorp, Warszawa, Poland) and were adjusted to pH 5.8 before autoclaving. Cultures were maintained at 25 ± 2°C in the dark. In 2010, a subset of ovules was subjected to heat shock (35°C) for 48 h immediately after excision.

After 6–8 wk on culture, the developing embryos and calli were transferred to R medium consisting of MS micro- and macroelements including vitamins, 40 μM glycine, 20 g l^−1^ sucrose, and 7 g l^−1^ Lab-agar, pH 5.8. Shoots were rooted on R medium supplemented with 0.1 mM IAA. Cultures were kept at 25 ± 2°C (16 h d, 55 μmol m^−2^ s^−1^) and were transferred to fresh media every 4 wk until plantlets with 4–5 leaves and roots were developed. The number of regenerated plants was recorded after 8 wk from the first transfer to the regeneration medium. Efficiency of regeneration was expressed as the number of obtained plants divided by the number of embryos and calli plated on R medium multiplied by 100%.

The ploidy level of regenerants was determined by flow cytometry as described by Kiełkowska and Adamus (2010). All diploid plants were then screened for PGI isozyme variants and DNA polymorphism at *chs2* and *ipi3* loci according to the protocols used for evaluation of the ovule donor plants.

## Statistical Analysis

Data were analyzed with ANOVA and mean separation was done according to Fisher’s LSD test at α = 0.05. Student *t* test was used to test significant differences in the frequency of the development of embryos and calli obtained from the ovules isolated from umbels pollinated with parsley pollen (PS and PU). Additionally, in unpollinated controls (UU and US), an upper confidence interval (CI_+95%_) for the mean number of embryo and callus development was calculated at α = 0.05.

## Results

### Seed set.

Six wk after pollination, the umbels kept on plants for natural seed setting were harvested. Except for five seeds on umbels pollinated with parsley pollen and treated with 2,4-D (PS), no seeds were found. These seeds were dried, stored for 6 mo at 4°C, and sown in pots, but they did not germinate over an observation period of 2 mo. Therefore, seeds set spontaneously or *via* induction by pollination with parsley pollen and 2,4-D treatment were not viable.

### In vitro development of ovules excised from unpollinated umbels.

One to three wk after pollination, ovules were excised from enlarged carrot ovaries derived from treatments PS and PU, and ovules of unpollinated umbels derived from treatments UU and US were excised and cultured *in vitro*. The UU and US control ovules enlarged *in vitro* and developed into embryos or calli with a low frequency independently of whether they came from 2,4-D-treated or untreated umbels (*p* = 0.34; Table 1). In 2008–2010, the percentage of developing ovules ranged from 0 to 0.46%, depending on the donor plant (Table 2), with the mean of all accessions of 0.11%. The upper 95% confidence interval (CI_+95%_) was 0.50%. Thus, any development observed in this work with a frequency higher than 0.50% was considered as induced by pollination.

**Table 1.** Mean percentage of ovules, developing embryos, or calli *in vitro* depending on pollination of carrot donor plants with parsley pollen and 2,4-D treatment (2008–2010)

Treatment	Pollination	2,4-D treatment	No. of plated ovules	Mean percentage of ovules developing			
				Embryos		Calli	
PS	Yes	Yes	33,296	1.43*	a	0.97*	a
PU	Yes	No	32,932	1.20*	a	0.18	b
US	No	Yes	5,780	0.05	b	0.22	b
UU	No	No	17,060	0.07	b	0.13	b

*Means exceeding 0.50%, *i.e.*, 95% upper confidence interval for unpollinated control

Means followed by the same *letters* in a *column* do not differ significantly according to Fisher’s LSD test at *p* = 0.05

**Table 2.** Mean percentage of ovules, developing embryos, or calli *in vitro* depending on carrot donor plant, pollination with parsley pollen, and 2,4-D treatment

Year	Accession	No. of plated ovules	Mean percentage of ovules developing							
			Embryos				Calli			
			PS	PU	UU	US	PS	PU	UU	US
2008	1/08	4,100	0.52*	0.25	0.00	0.00	0.26	0.00	0.00	0.00
	3/08	1,682	0.00	2.59*	0.00	0.00	0.00	0.00	0.00	0.00
	7333	2,820	0.10	0.71*	0.00	0.00	0.59*	0.00	0.00	0.00
	Karotan	2,520	0.07	1.05*	0.25	0.00	0.00	0.00	0.25	0.00
2009	1/09	2,800	0.00	1.25*	–	–	0.07	0.44	–	–
	2/09	3,360	0.27	0.26	–	–	0.09	0.17	–	–
	3/09	7,366	0.19	0.11	0.38	0.26	0.08	0.00	0.46	0.38
	8375	4,660	0.00	0.09	–	–	0.00	0.00	–	–
	8389	8,580	0.40	0.16	0.06	0.00	0.06	0.71*	0.00	0.30
	F1/09	8,600	0.00	0.08	0.06	0.00	1.16*	0.28	0.00	0.19
	Floret	2,440	0.05	0.00	–	–	0.16	0.32	–	–
	LB	4,280	0.00	3.29*	–	–	0.10	0.21	–	–
2010	POL 10/09	4,520	0.00	0.00	0.00	–	0.00	0.00	0.25	–
	POL 16/09	5,860	0.00	1.65*	0.13	–	0.09	0.09	0.00	–
	POL 5/09	4,980	0.17	5.39*	0.00	–	1.03*	0.09	0.33	–
	9490	3,720	9.55*	1.80*	0.00	–	2.76*	0.25	0.00	–
	9491	4,100	3.75*	0.32	0.00	–	3.83*	0.00	0.00	–
	01–09	3,560	0.87*	0.80*	0.00	–	0.56*	0.00	0.08	–
	02–09	3,620	3.47*	0.00	0.00	–	1.84*	0.56*	0.09	–
	11–09	5,500	3.29*	3.51*	0.19	–	3.01*	0.20	0.00	–

*Means exceeding 0.50%, *i.e.*, 95% upper confidence interval for unpollinated control

### Effect of accession, 2,4-D treatment, and time of ovule excision.

In general, pollination increased the frequency of ovule development. The mean percentage of developing ovules exceeded the CI_+95%_ by 2- to 3-fold for the control treatments, UU and US (Table 1). There was no significant effect of 2,4-D treatment applied after pollination on the frequency of the developing embryos, but 2,4-D stimulated callus development from cultured ovules 5-fold relative to callus development on untreated umbels (Table 1).

Accessions differed in their response to pollination and 2,4-D treatment. From 15 of 20 accessions, cultured ovules developed into embryos or calli more frequently than CI_+95%_ of the control. One accession (POL 10/09) did not respond at all to applied treatments, while ovules of the remaining four accessions did not respond differently than the unpollinated control (Table 2). In the accession with the highest response frequency (No. 9490), 9.55% of ovules developed into embryos. The next most responsive five accessions showed embryo development frequencies of 3.29 to 5.39%. Application of 2,4-D after pollination increased overall frequency of ovule development, but mainly by increasing the frequency of callus production.

The time after pollination of ovule excision affected development. Ovules excised 7 DAP showed very low response frequencies. Ovules excised 20–22 DAP developed embryos threefold more frequently than ovules excised 14–16 DAP (Table 3). The frequency of callus development did not depend on the age of the ovule.

**Table 3.** Mean percentage of ovules, developing embryos, or calli *in vitro* depending on the time of ovule excision from the ovary in 2009–2010. Unpollinated controls (UU and US) are excluded

Time of ovule excision [days after pollination]	No. of plated ovules	Mean percentage of ovules developing			
		Embryos		Calli	
7	12,186	0.09	c	0.34	a
14–16	20,980	1.02	b	0.86	a
20–22	11,420	2.99	a	0.73	a

Means followed by the same *letters* in a *column* do not differ significantly according to Fisher’s LSD test at *p* = 0.05

### Effect of induction media and heat shock.

The effect of medium composition was assessed separately for each year as different sets of accessions were used in each year (Table 1). All seven media induced ovule responses *in vitro* (Table 4). No significant differences in the frequencies of embryo and callus development were observed in response to culture on either full-strength or 1/2-strength MS media. The addition of 4.16 mM Edamin K (O medium) or 1 μM putrescine (HP, SHP medium) did not increase the frequency of embryo development. The addition of putrescine to the H medium, however, led to higher frequencies of callus production but not when used together with a higher level of IAA (0.57 μM) and with kinetin and GA_3_ (SHP medium). The addition of 9.08 μM TDZ influenced both embryo and callus development. The highest percentage of embryos (2.14%) was observed on the H medium supplemented with TDZ; however, on this medium, also the highest frequency of callus formation (1.69%) was observed. In all 3 yr, the use of medium H with 0.06 μM IAA allowed production of embryos at the same frequency as other media, while the frequency of callus development was reduced.

**Table 4.** Mean percentage of ovules, developing embryos, or calli *in vitro* depending on medium composition. Unpollinated controls (UU and US) are excluded

Year	Symbol	Medium		No. of plated ovules	Mean percentage of ovules developing			
		Minerals, vitamins	Supplement		Embryos		Calli	
2008	H	1/2 MS	0.06 μM IAA	7,160	0.15	a	0.07	a
	O	1/2 MS	4.16 mM Edamin K	6,182	0.48	a	0.24	a
2009	H	1/2 MS	0.06 μM IAA	9,695	0.36	a	0.15	bc
	HP	1/2 MS	0.06 μM IAA 1 mM putrescine	8,231	0.46	a	0.47	a
	SHI	MS	0.57 μM IAA 1.44 μM GA_3_ 4.60 μM KIN	8,520	0.49	a	0.22	b
	SHP	MS	0.57 μM IAA 1.44 μM GA_3_ 4.60 μM KIN 1 mM putrescine	8,880	0.32	a	0.03	c
2010	H	1/2 MS	0.06 μM IAA	9,020	1.70	ab	0.01	c
	HT	1/2 MS	0.06 μM IAA 9.08 μM TDZ	7,260	2.14	a	1.69	a
	SHI	MS	0.57 μM IAA 1.44 μM GA_3_ 4.60 μM KIN	7,840	1.42	ab	0.32	b
	SHT	MS	0.57 μM IAA 1.44 μM GA_3_ 4.60 μM KIN 9.08 μM TDZ	8,760	1.07	b	0.53	b

Means followed by the same *letters* in a *column* within each year do not differ significantly according to Fisher’s LSD test at *p* = 0.05

Heat shock of 35°C for 48 h applied to excised ovules *in vitro* decreased response frequencies. Heat-treated ovules developed embryos and calli almost threefold less frequently, in comparison to ovules kept at 25°C throughout the culture period (Table 5).

**Table 5.** Effect of heat shock on embryo and callus formation from ovules *in vitro*. Unpollinated controls (UU and US) are excluded

Temperature	No. of plated ovules	Mean percent of ovules developing			
		Embryos		Calli	
20–22°C	22,980	1.91	a	0.78	a
35°C for 48 h	12,880	0.58	b	0.25	b

Means in a *column* differ significantly according to Fisher’s LSD test at *p* = 0.05

### Validation of the optimal set of treatments and culture conditions.

The experiments conducted from 2008 to 2010 indicated optimal donor plant treatments and culture conditions that favored embryo development from excised ovules cultured *in vitro*. The highest frequency of response was obtained from ovules isolated 20–22 DAP from umbels not treated with 2,4-D (treatment PU). Culturing of excised ovules on H medium with 0.06 μM IAA at 25°C in the dark in 2010 resulted in a lack of callus development, while embryo development was obtained from six of eight accessions (Table 6). The frequency of embryo development in five responsive accessions ranged from 2.22 to 8.33%, and only one accession developed embryos at a low frequency (0.71%). The same set of treatments and conditions applied to an additional ten accessions in 2011 was found to be also advantageous in promoting embryo development as only two accessions produced callus at a low frequency (0.1%). In 2011, the efficiency of embryo development was high and, for seven accessions, varied from 1.38 to 11.98%. For one accession (No. 271), over 24% of ovules responded by embryo formation.

**Table 6.** Mean percentage of ovules, developing embryos, or calli *in vitro* depending on carrot donor plant in optimal conditions. Donors were pollinated with parsley pollen, not treated with 2,4-D. Ovules were excised 20–22 DAP and cultured on H medium at 20–22°C

Year	Accession	No. of plated ovules	Mean percentage of ovules developing	
			Embryos	Calli
2010	POL 10/09	960	8.33	0.00
	POL 16/09	860	0.00	0.00
	POL 5/09	920	2.22	0.00
	9490	800	6.25	0.00
	9491	760	0.71	0.00
	01–09	840	2.50	0.00
	02–09	680	0.00	0.00
	11–09	920	5.00	0.00
2011	271	980	24.29	0.10
	315	960	11.98	0.00
	348	1,060	9.72	0.00
	351	1,520	1.38	0.00
	845	940	0.11	0.00
	849	860	0.58	0.00
	850	1,120	11.25	0.00
	10–25	500	1.80	0.00
	38/10	980	3.67	0.10
	100/10	1,260	1.98	0.00

### Plant regeneration and characterization.

The embryos obtained from ovules cultured *in vitro* usually developed into individual plants after 3–4 transfers to fresh medium. When callus was used for plant production, plant recovery was considerably longer. The mean regeneration frequency over the years 2008–2010 was similar and ranged from 53.8 to 57.1% (Table 7). In 2011, the regeneration efficiency was much higher (82.8%), as no callus was produced and embryos were exclusively used for regeneration.

**Table 7.** Efficiency of plant regeneration from embryos and calli obtained from carrot ovules excised after pollination with parsley pollen

Year	Number of embryos and calli	Regenerated plants	
		Number	%
2008	56	32	57.1
2009	251	139	55.4
2010	625	336	53.8
2011	686	568	82.8

In each year of the study (2008–2011), the majority of plants obtained (78.2–99.1%) were diploids. Haploids (0.0–21.1%), tetraploids (0.0–2.6%), and mixoploids (0.0–0.9%) were also observed. Diploid plants were validated for homozygosity by isozyme and PCR analyses. Homozygosity varied depending on the accession and ranged from 0 up to 100% at all three loci (*pgi*-*2*, *ipi3*, and *chs2*) evaluated (data not shown). In total, from 2008 to 2011, 50.8% of embryo-derived and 16.5% of callus-derived diploid regenerants were homozygous. These results, considering haploids and embryo-derived homozygotic diploids jointly, showed that 72.6% of plants were of gametic origin.

## Discussion

Despite the widespread use of carrots as a model plant for cell and tissue cultures *in vitro*, the production of haploids and doubled haploids of carrots remains challenging. Carrot haploids can be obtained *via* induction of androgenesis or parthenogenesis, although the efficiency of these processes was low (Matsubara et al. 1995, Adamus and Michalik 2003, Li et al. 2013). Kiełkowska and Adamus (2010) showed that several species can be used as pollen sources to induce parthenogenetic development in carrots, with parsley being the most effective pollen source. Parsley does not cross with carrots; however, its pollen germinates on the carrot stigma and induces ovule development. Wide pollination with parsley pollen and carrot ovule isolation were both essential for ovule development and plant recovery. Unpollinated flowers did not set seeds spontaneously, and the few seeds produced after pollination and 2,4-D treatment were not viable. The development of ovules isolated from unpollinated umbels was low and did not exceed 0.5%.

A proper time of ovule excision was important for embryo development *in vitro*. The optimal time of isolation was species-dependent. The highest embryogenic response of cotton ovules stimulated to develop by hibiscus pollen was obtained from ovules isolated 10 DAP and no response was observed in ovules isolated 3 DAP (Kantartzi and Roupakias 2009). For durum wheat pollinated with maize, the optimal time for embryo rescue was 12–15 DAP (Cherkaoui et al. 2000) or 18 DAP (Sourour et al. 2012). In snapmelon pollinated with irradiated pollen, haploid plants were obtained only from embryos excised 21 DAP (Godbole and Murthy 2012). In the current study, ovules were excised from the ovaries and cultured on the media in 1-wk intervals starting 7 DAP and ending 20–22 DAP. The highest response was observed from ovules isolated 20–22 DAP. Shortening this time by 1 wk reduced the efficiency over threefold. The majority of ovules excised 20–22 DAP developed into embryos, from which plants could be easily regenerated. From younger ovules, not only was the efficiency lower, but also a higher frequency of undesired callus formation was observed.

The application of plant growth regulators on inflorescences of the female parent of interspecific or intergeneric crosses promoted the development of haploid embryos. Such treatment was beneficial for wheat × maize (Brazauskas et al. 2004), maize × sorghum/pearl millet (Manickam and Sarkar 1999), and triticale × maize (Wędzony et al. 1998) crosses, but had no effect in a cotton × hibiscus (Kantartzi and Roupakias 2009) cross. In carrots, the post-pollination application of 2,4-D stimulated foreign pollen tube growth in pistils and significantly increased the number of developing ovules in comparison to 2,4-D-untreated control (Kiełkowska and Adamus 2010). The use of 450 μM 2,4-D in this study increased the frequency of ovule response, but stimulated the development of unorganized callus tissue rather than embryo formation. Although callus may develop from haploid cells, more often it results of somatic cell proliferation (Thomas et al. 2003, Staniaszek and Habdas 2006) and is undesired in haploid production. Additionally, callus is a genetically unstable tissue in which a high degree of genetic variation can occur (Ghosh et al. 2010) resulting also in altered ploidy of regenerated plants (Larkin and Scowcroft 1981). In this light, the use of pollination without 2,4-D treatment was better for direct production of carrot haploid and DH plants.

Culture media was important for successful ovule culture and embryo rescue. The addition of Edamin to a 1/2-strength MS medium favored cucumber haploid embryo development and storage in a long-term culture (Niemirowicz-Szczytt et al. 2000). Edamin is a product of enzymatic digestion of lactalbumin, contains amino acids and peptides, and is a source of organic nitrogen (Northey and Brooks 1962). Polyamines like spermidine or putrescine improved induction of gynogenic embryo formation and regeneration of haploid plantlets (Martinez et al. 2000, Ebrahimi and Zamani 2009). Thidiazuron is another growth regulator widely used as supplement of induction and regeneration media for improving gynogenic response (Chen et al. 2011). TDZ was successfully used for induction of cucumber gynogenic embryos and plant regeneration (Gemes-Juhasz et al. 2002; Diao et al. 2009). In this study, we used several media differing in their composition and containing active compounds listed above and also others like kinetin and gibberellic acid. None of these components stimulated ovules to produce embryos more effectively than 0.06 μM IAA.

Heat shock may be an effective trigger in switching the gametophytic development of microspore to the sporophytic pathway as shown in *Brassica* microspore culture (Duijs et al. 1992, Dias 2001). In cucumber ovary culture, the application of 3-d heat shock of 35°C stimulated embryo formation (Diao et al. 2009). In red beet, culture at 27°C and 32°C for 48 h doubled the number of responding ovules in comparison to the control kept at 25°C (Baranski 1996). Contrary to those reports, we found that heat shock had an adverse effect on carrot ovules when applied in the first 48 h of culture.

The genotype of the donor plant is one of the most important factors affecting haploid plant induction regardless of the methodology used (Phippen and Ockendon 1990, Martinez et al. 2000, Chen et al. 2011). This is the first evaluation of such a wide range of accessions that has resulted in a high frequency of embryo production from the female gametophyte. In total, 30 different carrot accessions were used, and the effect of accession was clearly observable. We were able to select a set of treatments and culture conditions that were most advantageous for carrot embryo development. This procedure required pollination of carrot umbels with parsley pollen, further donor plant growth for the next 3 wk without any 2,4-D treatment, and then excision of the ovules and their subsequent culture *in vitro* using a 1/2-strength MS medium containing 0.06 μM IAA at 25°C in the dark. Using this procedure, most donors responded with embryo development at a frequency of 1–12%, while formation of undesired callus was very limited and did not exceed 0.1%. The usefulness of this procedure was confirmed in experiments carried out in two consecutive years with 18 different carrot accessions, and 15 responded by producing embryos exclusively. Moreover, we identified a superior accession (No. 271) that was highly responsive to optimized applied conditions. Li et al. (2013) cultured microspores of 47 carrot accessions and reported that 28 of them responded by producing either callus (11 accessions) or callus and embryos (17 accessions), and none of these accessions produced embryos exclusively. In contrast, in our protocol, a high proportion of embryos was observed. Moreover, these embryos had a higher ability to regenerate into plants compared to callus, which confirmed observations made after androgenesis by Matsubara et al. (1995).

The majority of obtained plants were haploids or diploids. Diploids were identified as homozygous at three independent loci, *pgi*-*2*, *chs2*, and *ipi3*. Considering that the donor plants used were heterozygous at these loci, the obtained diploids probably developed from cells of gametic origin and spontaneous chromosome doubling resulted in the production of doubled haploid plants. Spontaneous diploidization was observed previously in carrots (Monakhova et al. 1998, Shmykova and Tyukavin 2002, Li et al. 2013). The protocol described here enabled production of either DHs or haploid plants from carrot female gametophytes. Such plants can be utilized for further research or after diploidization for breeding purposes.

## References

[CR1] Adamus A, Michalik B (2003). Anther cultures of carrot (*Daucus carota* L.). Folia Hortic.

[CR2] Baranski R (1996). In vitro gynogenesis in red beet (*Beta vulgaris* L.): effects of ovule culture conditions. Acta Soc Bot Pol.

[CR3] Baranski R (2000). Application of rapid glucose-6-phosphate isomerase assay in white cabbage breeding. Acta Physiol Plant.

[CR4] Baranski R, Allender C, Klimek-Chodacka M (2012). Towards better tasting and more nutritious carrots: carotenoid and sugar content variation in carrot genetic resources. Food Res Int.

[CR5] Bohanec B, Jakše M, Ihan A, Javornik B (1995). Studies of gynogenesis in onion (*Allium cepa* L.): induction procedures and genetic analysis of regenerants. Plant Sci.

[CR6] Brazauskas G, Pasakinskiene I, Jahoor A (2004). AFLP analysis indicates no introgression of maize DNA in wheat x maize crosses. Plant Breed.

[CR7] Chen J-F, Cui L, Malik AA, Mbira KG (2011). In vitro haploid and dihaploid production via unfertilized ovule culture. Plant Cell Tissue Organ Cult.

[CR8] Cherkaoui S, Lamsaouri O, Chlyah A, Chlyah H (2000). Durum wheat×maize crosses for haploid wheat production: influence of parental genotypes and various experimental factors. Plant Breed.

[CR9] Diao WP, Jia YY, Song H, Zhang XQ, Lou QF, Chen JF (2009). Efficient embryo induction in cucumber ovary culture and homozygous identification of the regenerants using SSR markers. Sci Hortic.

[CR10] Dias JS (2001). Effect of incubation temperature regimes and culture medium on broccoli microspore culture embryogenesis. Euphytica.

[CR11] Duijs JG, Voorrips RE, Visser DL, Custers JBM (1992). Microspore culture is successful in most crop types of *Brassica oleracea* L. Euphytica.

[CR12] Ebrahimi R, Zamani Z (2009). Effect of polyamines on in vitro gynogenesis of onion (*Allium Cepa* L.). Am Eurasian J Sustain Agric.

[CR13] Eenink AH (1974). Matromorphy in *Brassica oleracea* L. I. Terminology, parthenogenesis in Cruciferae and the formation and usability of matromorphic plants. Euphytica.

[CR14] FAO (2010) http://faostat.fao.org/faostat/10.10.2012

[CR15] Foroughi-Wehr B, Wenzel G, Hayward WD, Bosemark NO, Romagosa I (1993). Andro- and parthenogenesis. Plant breeding: principles and prospects.

[CR16] Gamborg OL, Miller RA, Ojima O (1968). Nutrient requirements of suspension cultures of soybean root cell. Exp Cell Res.

[CR17] Gemes-Juhasz A, Balogh P, Ferenczy A, Kristof Z (2002). Effect of optimal stage of female gametophyte and heat treatment on in vitro gynogenesis induction in cucumber (*Cucumis sativus* L.). Plant Cell Rep.

[CR18] Ghosh N, Smith DW, Das AB, Chatterjee A (2010). Rapid in vitro regeneration and clonal propagation of the fastest growing leguminous tree *Albizia falcataria* (L.) Fosberg using leaflet explant. Plant Tissue Cult Biotechnol.

[CR19] Godbole M, Murthy HN (2012). Parthenogenetic haploid plants using gamma irradiated pollen in snapmelon (*Cucumis melo* var. *momordica*). Plant Cell Tissue Org Cult.

[CR20] Kantartzi SK, Roupakias G (2009). In vitro gynogenesis in cotton (Gossypium sp.). Plant Cell Tissue Org Cult.

[CR21] Kiełkowska A, Adamus A (2010). In vitro culture of unfertilized ovules in carrot (*Daucus carota* L.). Plant Cell Tissue Org Cult.

[CR22] Larkin PJ, Scowcroft WR (1981). Somaclonal variation— a novel source of variability from cell cultures for plant improvement. Theor Appl Genet.

[CR23] Li J, Li J-R, Zhuang F-Y, Ou C-G, Hu H, Zhao Z-W, Mao J–H (2013). Microspore embryogenesis and production of haploid and doubled haploid plants in carrot (*Daucus carota* L.). Plant Cell Tissue Org Cult.

[CR24] Manickam S, Sarkar KR (1999). Foreign pollen tube growth in maize after chemical treatments. Indian J Genet Plant Breed.

[CR25] Martinez LE, Aguero CB, Lopez ME, Galmarini CR (2000). Improvement of in vitro gynogenesis induction in onion (*Allium cepa* L.) using polyamines. Plant Sci.

[CR26] Matsubara S, Dohya N, Murakami K (1995). Callus formation and regeneration of adventitious embryos from carrot, fennel and mitsuba microspores by anther and isolated microspore cultures. Acta Horticult.

[CR27] Monakhova MA, Tyukavin GB, Shmykova NA (1998) Change of ploidy during formation regenerated plants from androgenous and gynogenous embryos of carrot in vitro. In: Proceedings of the IX International Congress on Plant Tissue and Cell Culture, Plant biotechnology and in vitro biology in the 21st century. Jerusalem, Israel, p 122

[CR28] Murashige T, Skoog F (1962). A revisedmediumfor rapid growth and bioassays with tobacco tissue cultures. Physiol Plant.

[CR29] Niemirowicz-Szczytt K, Faris NM, Rucińska M, Nikolova V (2000). Conservation and storage of a haploid cucumber (*Cucumis sativus* L.) collection under in vitro conditions. Plant Cell Rep.

[CR30] Northey WT, Brooks LD (1962). Studies on Coccidioides immits I. A simple medium for in vitro spherulation. J Bacteriol.

[CR31] Phippen CI, Ockendon DJ (1990). Genotype, plant, bud size and factors affecting anther culture of cauliflower (*Brassica oleracea* var. *botrytis*). Theor Appl Genet.

[CR32] Rogers SO, Bendich AJ, Gelvin SB, Schilperoort RA (1988). Extraction of DNA from plant tissues. Plant molecular biology manual.

[CR33] Rubatzky VE, Quiros CF, Simon PW (1999). Carrots and related vegetable Umbelliferae.

[CR34] Sacks EJ (2008). Ovule rescue efficiency of Gossypium hirsutum×G. arboreum progeny from field-grown fruit is affected by media composition and antimicrobial compounds. Plant Cell Tissue Org Cult.

[CR35] Shmykova NA, Tyukavin GB (2002). Characteristics of morphological and genetic changes during in vitro culturing of carrot anthers. Biotechnology (Биотехнология).

[CR36] Simon PW, Freeman RE, Vieira JV, Boiteux LS, Briard M, Nothnagel T, Michalik B, Kwon Y, Prohens J, Nuez F (2008). Carrot. Handbook of plant breeding.

[CR37] Sourour A, Zoubeir C, Ons T, Youssef T, Hajer SA (2012). Performance of durum wheat (*Triticum durum* L.) doubled haploids derived from durum wheat×maize crosses. J Plant Breed Crop Sci.

[CR38] Staniaszek M, Habdas H (2006). RAPD technique application for intraline evaluation of androgenic carrot plants. Folia Hortic.

[CR39] Stein M, Nothnagel T (1995). Some remarks on carrot breeding (*Daucus carota sativus* Hoffm.). Plant Breed.

[CR40] Thomas WTB, Forster B, Gertsson B, Maluszynski M, Kasha KJ, Forster BP, Szarejko I (2003). Doubled haploids in breeding. Doubled haploids production in crop plants.

[CR41] Virk DS, Gupta AK (1984). Matromorphy in Pisum sativum L. Theor Appl Genet.

[CR42] Wędzony M, Marcińska I, Ponitka A, Ślusarkiewicz-Jarzina A, Wozna J (1998). Production of doubled haploids in triticale (×*Triticosecale*Wittm.) by means of crosses with maize (*Zea mays* L.) using picloram and dicamba. Plant Breed.

